# Supplementary material for: Different Distribution Patterns of Hoverflies (Diptera: Syrphidae) and Bees (Hymenoptera: Anthophila) Along Altitudinal Gradients in Dolomiti Bellunesi National Park (Italy)
Source: Insects. 2022 Mar 15;13(3):293. doi: 10.3390/insects13030293 (PMC8950664; doi:10.3390/insects13030293)
Supplement: Supplementary file 1 [file insects-13-00293-s001.zip › insects-1603875-supplementary.pdf]

Table S1. Detailed of the three specimens identified with barcoding. The Accession Number is from GenBank where the nucleotide sequences have been deposited. For more detail about the Collection Locality see Tab. 1.

**Species**	**Sex**	**Collection Locality**	**Date**	**Altitude**	**Accession number**
Psithyrus bohemicus	Female	Soladen VFB	13.ix.2020	956	OM836488
Bombus wurflenii	Female	Vette Grandi VFH	13.ix.2020	2130	OM836489
Bombus soroeensis	Female	Soladen VFB	13.ix.2020	956	OM836490

Table S2. Correlation matrix between predicting variables. The table reports the P value of Sperman linear correlation. In bold the statistically significant correlation ($P < 0.05$). abbreviation: Alt = Altitude; Grass = Percentage of grassland habitat; N Sp Ent = number of plant species with enotomophilic pollination; N Sp Veg = number of plant species; Scree = percentage of scree area; Scrubs = percentage of scrubs habitat; Temp = temperature; Wood = Percentage of wood habitat. See text fo a more detailed description of the predicting variables.

	Temp	Wood	Grass	Scree	Scrubs	Slope	N Sp Veg	N Sp Ent
Alt	**0.0000**	0.2243	0.9235	**0.0275**	0.8062	0.0651	0.9368	0.4421
Temp		0.2048	0.8109	0.1227	0.9136	0.1245	0.8847	0.7436
Wood			**0.0342**	0.2896	0.8214	0.3049	0.7919	0.7222
Grass				0.2038	**0.0475**	0.6612	0.7047	0.8466
Scree					0.4246	0.2696	0.2042	0.8121
Scrubs						0.0569	0.2042	0.0732
Slope							0.3036	**0.0305**
N Sp Veg								**0.0002**

Table S3. List of Syrphidae species collected by pan traps, with the main ecological categories used in the analysis. Abbreviations: Fol. = Foliage; Sap. = Saprophagous; Xylosap. = Xylosaprophagous. For Adult Dimension the following categories have been used: small = 6-9 cm; medium = 9.5-12 cm, large > 12 cm.

**Species**	**Larvae Diet**	**Larvae Microhabitat**	**Adult Dimension**
Brachypalpoides lentus (Meigen, 1822)	Xylosap.	Wood	Large
Brachypalpus chrysites Egger, 1859	Xylosap.	Wood	Large
Cheilosia aerea Dufour, 1848	Phytophagous	Herb, Root	Small
Cheilosia canicularis (Panzer, 1801)	Phytophagous	Herb, Root	Large
Cheilosia carbonaria Egger, 1860	Phytophagous	Herb, Root	Medium
Cheilosia derasa Loew, 1857	Phytophagous	Herb, Root	Small
Cheilosia gagatea Loew, 1857	Phytophagous	Herb	Medium
Cheilosia grisella Becker, 1894	Phytophagous	Herb, Root	Small
Cheilosia illustrata (Harris, 1776)	Phytophagous	Herb, Root	Medium
Cheilosia impressa Loew, 1840	Phytophagous	Herb, Root	Small

Cheilosia laeviseta Claussen, 1987	Phytophagous	Herb, Root	Small
Cheilosia laticornis Rondani, 1857	Phytophagous	Herb, Root	Medium
Cheilosia pagana (Meigen, 1822)	Phytophagous	Herb, Root	Small
Cheilosia personata Loew, 1857	Phytophagous	Herb	Medium
Cheilosia rhynchops Egger, 1860	Phytophagous	Herb, Root	Medium
Cheilosia vernalis (Fallén, 1817)	Phytophagous	Herb, Root	Small
Chrysotoxum bicinctum (Linnaeus, 1758)	Zoophagous	Root	Medium
Chrysotoxum elegans Loew, 1841	Zoophagous	Root	Medium
Chrysotoxum fasciatum (Müller, 1764)	Zoophagous	Root	Medium
Dasysyrphus albostriatus (Fallén, 1817)	Zoophagous	Tree Fol.	Medium
Epistrophe grossulariae (Meigen, 1822)	Zoophagous	Herb	Medium
Episyrphus balteatus (De Geer, 1776)	Zoophagous	Herb, Tree Fol.	Medium
Eristalis arbustorum (Linnaeus, 1758)	Water Sap.	Water	Medium
Eristalis interrupta (Poda, 1761)	Water Sap.	Water	Medium
Eristalis pertinax (Scopoli, 1763)	Water Sap.	Water	Large
Eristalis similis (Fallén, 1817)	Water Sap.	Water	Large
Eristalis tenax (Linnaeus, 1758)	Water Sap.	Water	Large
Eumerus alpinus Rondani, 1857	Phytophagous	Herb, Root	Medium
Eumerus ornatus Meigen, 1822	Phytophagous	Herb, Root	Small
Eumerus sinuatus Loew, 1855	Phytophagous	Herb, Root	Medium
Eupeodes corollae (Fabricius, 1794)	Zoophagous	Tree Fol.	Small
Eupeodes lapponicus (Zetterstedt, 1838)	Zoophagous	Tree Fol.	Medium
Eupeodes luniger (Meigen, 1822)	Zoophagous	Tree Fol.	Medium
Ferdinandea cuprea (Scopoli, 1763)	Xylosap.	Wood	Medium
Melanostoma mellinum (Linnaeus, 1758)	Zoophagous	Herb, Root	Small
Meliscaeva auricollis (Meigen, 1822)	Zoophagous	Tree Fol.	Medium
Meliscaeva cinctella (Zetterstedt, 1843)	Zoophagous	Herb, Root	Medium
Merodon aeneus Meigen, 1822	Phytophagous	Herb, Root	Small
Merodon armipes Rondani, 1843	Phytophagous	Herb, Root	Medium
Merodon cinereus (Fabricius, 1794)	Phytophagous	Herb, Root	Medium
Merodon constans (Rossi, 1794)	Phytophagous	Herb, Root	Medium
Merodon equestris (Fabricius, 1794)	Phytophagous	Herb, Root	Large
Merodon rufus Meigen, 1838	Phytophagous	Herb, Root	Medium
Microdon devius (Linnaeus, 1761)	Zoophagous	Root	Medium
Myathropea florea (Linnaeus, 1758)	Water Sap.	Water	Large
Paragus pecchiolii Rondani, 1857	Zoophagous	Herb	Small
Pipiza noctiluca (Linnaeus, 1758)	Zoophagous	Herb	Small
Platycheirus albimanus (Fabricius, 1781)	Zoophagous	Herb, Tree Fol.	Small
Platycheirus manicatus (Meigen, 1822)	Zoophagous	Herb	Small
Platycheirus nielseni Vockeroth, 1990	Zoophagous	Herb, Tree Fol.	Small
Platycheirus scutatus (Meigen, 1822)	Zoophagous	Herb, Tree Fol.	Small
Rhingia campestris Meigen, 1822	Water Sap.	Water	Medium

Scaeva dignota (Rondani, 1857)	Zoophagous	Herb, Tree Fol.	Large
Scaeva pyrastri (Linnaeus, 1758)	Zoophagous	Herb, Tree Fol.	Large
Sphaerophoria fatarum Goeldlin de Tiefenau, 1974	Zoophagous	Herb	Small
Sphaerophoria interrupta (Fabricius, 1805)	Zoophagous	Herb	Small
Sphaerophoria scripta (Linnaeus, 1758)	Zoophagous	Herb	Small
Sphaerophoria taeniata (Meigen, 1822)	Zoophagous	Herb	Small
Syrphus ribesii (Linnaeus, 1758)	Zoophagous	Herb	Medium
Syrphus torvus Osten-Sacken, 1875	Zoophagous	Herb	Medium
Syrphus vitripennis Meigen, 1822	Zoophagous	Herb	Medium
Trichopsomyia joratensis (Goeldlin de Tiefenau, 1997)	Zoophagous	Herb	Small
Volucella bombylans (Linnaeus, 1758)	Zoophagous	Root	Large
Volucella pellucens (Linnaeus, 1758)	-	-	Large
Xylota ignava (Panzer, 1798)	Xylosap.	Wood	Large
Xylota jakutorum Bagatshanova, 1980	Xylosap.	Wood	Medium
Xylota segnis (Linnaeus, 1758)	Xylosap.	Wood	Medium
Xylota sylvarum (Linnaeus, 1758)	Xylosap.	Wood	Large

Table S4. List of Anthophila species collected by pan traps, with the main ecological categories used in the analysis. Abbreviations: Soc. = Social; Veg. = Vegetation. For Adult Dimension the following categories have been used: small = 5-10 cm; medium = 10.5-15.5 cm; large > 15.5 cm.

Specie	**Family**	**Nest Position**	**Diet Breath**	**Adult Dimension**
Andrena aeneiventris Morawitz, 1872	Andrenidae	Soil	Oligolectic	Small
Andrena bicolor Fabricius, 1775	Andrenidae	Soil	Polilectic	Small
Andrena cinerea Brullé, 1832	Andrenidae	Soil	Polilectic	Small
Andrena dorsata (Kirby, 1802)	Andrenidae	Soil	Polilectic	Small
Andrena fulvago (Christ, 1791)	Andrenidae	Soil	Polilectic	Medium
Andrena hattorfiana (Fabricius, 1775)	Andrenidae	Soil	Oligolectis	Medium
Andrena nana (Kirby, 1802)	Andrenidae	Soil	Polilectic	Small
Andrena ovatula (Kirby, 1802)	Andrenidae	Soil	Polilectic	Small
Andrena similis Smith, 1849	Andrenidae	Soil	Oligolectic	Medium
Andrena ventralis Imhoff, 1832	Andrenidae	Soil	Oligolectic	Small
Andrena vulpecula Kriechbaumer, 1873	Andrenidae	Soil	Oligolectic	Small
Andrena wilkella (Kirby, 1802)	Andrenidae	Soil	Oligolectic	Medium
Apis mellifera Linnaeus, 1758	Apidae	-	Polilectic	Medium
Bombus hortorum (Linnaeus, 1761)	Apidae	Soil	Polilectic	Medium
Bombus inexspectatus (Tkalcú, 1963)	Apidae	Soil	Soc. Parasite	Large
Bombus jonellus (Kirby, 1802)	Apidae	Soil	Oligolectic	Medium
Bombus lapidarius (Linnaeus, 1758)	Apidae	Soil	Polilectic	Medium
Bombus lucorum (Linnaeus, 1761)	Apidae	Soil	Polilectic	Large
Bombus pascuorum (Scopoli, 1763)	Apidae	Soil	Polilectic	Medium

Bombus pratorum (Linnaeus, 1761)	Apidae	Soil	Polilectic	Medium
Bombus ruderarius (Müller, 1776)	Apidae	Soil	Polilectic	Large
Bombus soroeensis (Fabricius, 1776)	Apidae	Soil	Soc. Parasite	Medium
Bombus sp 2	Apidae	-	-	-
Bombus terrestris (Linnaeus, 1758)	Apidae	Soil	Polilectic	Medium
Bombus wurfleini Radoszkowski, 1859	Apidae	Soil	Polilectic	Medium
Eucera nigrifacies Lepeletier, 1841	Apidae	Veg.	Polilectic	Medium
Halictus sajoi Blüthgen, 1923	Halictidae	Soil	Polilectic	Small
Halictus scabiosae (Rossi, 1790)	Halictidae	Soil	Polilectic	Medium
Halictus sexcintus (Fabricius, 1775)	Halictidae	Soil	Polilectic	Medium
Halictus tumulorum (Linnaeus, 1758)	Halictidae	Soil	Polilectic	Small
Hoplitis claviventris (Thompson, 1872)	Megachilidae	Veg.	Polilectic	Small
Hylaeus brevicornis Nylander, 1852	Colletidae	Veg.	Polilectic	Small
Hylaeus communis Nylander, 1852	Colletidae	Veg.	Polilectic	Small
Hylaeus glacialis Morawitz, 1872	Colletidae	Veg.	-	Small
Hylaeus hyalinatus Smith, 1842	Colletidae	Holes	Polilectic	Small
Lasioglossum albipes (Fabricuis, 1781)	Halictidae	Soil	Polilectic	Small
Lasioglossum calceatum (Scopoli, 1763)	Halictidae	Soil	Polilectic	Small
Lasioglossum fulvicorne (Kirby, 1802)	Halictidae	Soil	Polilectic	Small
Lasioglossum laevigatum (Kirby, 1802)	Halictidae	Soil	Polilectic	Small
Lasioglossum laticeps (Schenck, 1868)	Halictidae	Soil	Polilectic	Small
Lasioglossum malachurum (Kirby, 1802)	Halictidae	Soil	Polilectic	Small
Lasioglossum nitidulum (Fabricius, 1804)	Halictidae	Soil	Polilectic	Small
Lasioglossum pauxillum (Schenck, 1853)	Halictidae	Soil	Polilectic	Small
Lasioglossum punctatissimum (Schenck, 1853)	Halictidae	Soil	Polilectic	Small
Lasioglossum subhirtum (Lepeletier, 1841)	Halictidae	Soil	Polilectic	Small
Lasioglossum trunctaticolle (Morawitz, 1877)	Halictidae	-	-	-
Lasioglossum villosulum (Kirby, 1802)	Halictidae	Soil	Polilectic	Small
Lasioglossum zonulum (Smith, 1848)	Halictidae	Soil	Polilectic	Small
Megachile circumcincta Kirby, 1802	Megachilidae	Soil, Veg.	Polilectic	Medium
Megachile lagopoda (Linnaeus, 1761)	Megachilidae	Soil	Polilectic	Medium
Megachile pilicrus Morawitz, 1877	Megachilidae	Soil	Polilectic	Medium
Nomada alboguttata Herrich-Schäffer, 1839	Apidae	Soil	Soc. Parasite	Small
Osmia aurulenta Panzer, 1799	Megachilidae	Soil, Holes	Polilectic	Medium
Osmia bicolor (Schrank, 1781)	Megachilidae	Holes	Polilectic	Small
Osmia caerulescens (Linnaeus, 1758)	Megachilidae	Veg., Holes	Polilectic	Small
Osmia cerinthidis Morawitz, 1876	Megachilidae	Soil	Oligolectic	Medium
Osmia emarginata Lepeletier, 1841	Megachilidae	Holes	Polilectic	Medium
Osmia leaiana (Kirby, 1802)	Megachilidae	Veg., Holes	Oligolectic	Small

Osmia mustelina Gerstaecker, 1869	Megachilidae	Holes	Poligolectic	Small
Psithyrus campestris (Panzer, 1801)	Apidae	Soil	Soc. Parasite	Large
Psithyrus cfr *bohemicus* (Seidl, 1838)	Apidae	Soil	Soc. Parasite	Large
Psithyrus maxillosus (Klug, 1817)	Apidae	Soil	Soc. Parasite	Large
Psithyrus rupestris (Fabricius, 1793)	Apidae	Soil	Soc. Parasite	Large
Psithyrus sylvestris (Lepeletier, 1832)	Apidae	Soil	Soc. Parasite	Medium
Psithyrus vestalis (Geoffroy, 1785)	Apidae	Soil	Soc. Parasite	Large
Tetraloniella salicariae (Lepeletier, 1841)	Apidae	Veg.	Oligolectic	Small
Xylocopa violacea (Linnaeus, 1758)	Apidae	Veg.	Polilectic	Large
